# Exercise induced hypoalgesia after a high intensity functional training: a randomized controlled crossover study

**DOI:** 10.1186/s13102-024-00969-4

**Published:** 2024-08-28

**Authors:** Fabian Tomschi, Pia Ransmann, Alexander Schmidt, Thomas Hilberg

**Affiliations:** Department of Sports Medicine, University of Wuppertal, Wuppertal, Germany

**Keywords:** HIIT, High intensity interval training, Pain, Sensitivity, Sex-specific, Gender

## Abstract

**Background:**

Acute physical activity often induces an acute reduction in pain sensitivity known as exercise induced hypoalgesia (EIH). The aim of this study was to investigate the effects of a high intensity functional training (HIFT) on EIH compared to a control session.

**Methods:**

50 (age: 26.0 ± 2.7; 23 female) participants successfully conducted this study consisting of a pre-experimental test as well as a 12-minute HIFT (body-weight exercises) and a 12-minute control (supervised breathing) session in a randomized crossover design. Pre and post, pressure pain thresholds (PPT) were measured at the ankles, knees, elbows, and forehead.

**Results:**

The HIFT resulted in a relative maximum and average heart rate of 96.2% (± 3.6%) and 91.1% (± 4.2%), respectively, and maximum and average RPE values of 19.1 (± 1.2) and 16.2 (± 1.4), respectively. Results reveal a significant ‘Intervention’ × ‘Time point’ interaction (*p* < 0.001) for PPT (pooled for one average value) with hypoalgesia observed following the HIFT (*p* < 0.001; pre: 56.0 ± 16.8, post: 61.6 ± 19.0 [Newton]) and no change following the control (*p* = 0.067; pre: 56.6 ± 18.4, post: 55.3 ± 18.9 [Newton]). Further, a significant ‘Time’ × ‘Intervention’ × ‘Landmark’ interaction effect (*p* = 0.024) is observed and all landmarks showed significant hypoalgesia following HIFT (*p* < 0.01), except for the right elbow and forehead. Following control, no hypoalgesia was observed at any landmark. Analysing male and female participants separately, it was observed that EIH occured only in men.

**Conclusion:**

A HIFT using bodyweight exercises reduces pain sensitivity. Hence, combining strength and aerobically demanding exercises in a short but high intensity manner, as done in HIFT, can be seen as a usable tool to induce hypoalgesia. Yet, these results were observed only in male participants, necessitating future sex-specific research.

**Trial registration:**

DRKS00034391, retrospectively registered on the 4th of June 2024.

**Supplementary Information:**

The online version contains supplementary material available at 10.1186/s13102-024-00969-4.

## Introduction

Major components of health-related fitness (e.g., aerobic and strength capacity, body composition as well as cardiovascular, metabolic and mental health, etc.) can be improved by regular physical activity [1, 2]. Physical exercise further contributes to a reduction of overall bodily pain in the long term [3] as well as to a reduced pain sensitivity after exercise. This acute reduction in pain sensitivity is known as “exercise induced hypoalgesia” (EIH) [4, 5]. Pain sensitivity can be measured using different experimental stimuli (thermal, mechanical, chemical) while the use of mechanical pressure pain thresholds (PPT) is recommended and usually used in the context of EIH studies [6]. EIH studies in the healthy population have used a variety of exercise protocols in terms of exercise type, time, and intensity and it was demonstrated that aerobic exercise and resistance exercise can result in hypoalgesic effects [6]. However, these different exercise types seem to induce EIH in a different magnitude. A recent meta-analysis revealed that aerobic exercise results in largest EIH effects while dynamic resistance exercise only induces small EIH effects and isometric exercise does not induce any effects [7]. There is further evidence that higher intensity aerobic exercise protocols seem to induce higher EIH compared to more moderate and metabolically less demanding aerobic exercises [8]. Besides, available literature indicates that there might be variations in pain perception and EIH between male and female participants. Yet, these results are equivocal with studies indicating that exercise results in EIH in women but not in men [9, 10], while also no sex-dependent differences in EIH were reported [11]. Investigating these differences is essential to determine whether specific exercise regiments are equally effective for both males and females.

Recently, a new method to improve health-related fitness has emerged, namely the high intensity functional training (HIFT) [12]. HIFT can be characterized by possessing the intensity of high intensity interval training (HIIT) programs (along with the usually rather short duration), while incorporating functional strength and aerobically demanding movements. Usually, HIFT can be conducted with no or little equipment and can further be performed at home [13]. HIFT integrates a wider variety of exercises compared to the more conventional HIIT. HIIT usually consists of unimodal endurance-focused modalities (i.e., running, cycling, rowing, etc.). Contrastingly, HIFT aims at using multimodal and “functional” multi-joint strength training exercises involving mostly whole body, multiple planes movements. This can be performed using only ones own bodyweight e.g., squats, lunges, vertical jumps, and push-ups or with additional free weights, e.g., barbells, kettlebells, dumbbells, or medicine balls [14, 15]. Recent literature presents that HIIT and HIFT regiments both improve aerobic capacity and anaerobic power [16]. However, HIFT further induces improvements in muscle strength, power, and muscular endurance [17] as it provides more complex stimuli compared to the unimodal HIIT.

Due to the fact that HIFT incorporates both aerobically demanding exercises as well as strength exercises it presents a highly interesting novel training modality to explore pain physiological effects of this exercise model on EIH. To the best of our knowledge, available EIH-literature mostly explored unimodal exercise designs and its effects on EIH, while no study explored an exercise model that induced both strength and aerobic physiological stimuli of high intensity. Therefore, the aim of this study was to evaluate the potential hypoalgesic effects of a 12-minute HIFT program compared to a control session. Based on these considerations the following hypotheses were formulated: (1) One bout of HIFT results in an acute reduced pain sensitivity compared to a control session. (2) This hypoalgesia occurs primarily at the body landmarks close to majorly working musculature. (3) There is no difference in the hypoalgesic response between male and female participants.

## Methods

### General study design

The study was designed as a randomized controlled crossover trial and consisted of three sessions. All examinations were performed in the same laboratory of the Department of Sports Medicine (University of Wuppertal, Germany) from March 2022 to February 2024. First, a pre-experimental session was conducted, in which eligibility of the participants in terms of in- and exclusion criteria was determined, anthropometric data were obtained, and an incremental bicycle ergometer test up to exhaustion was performed. In the two subsequent sessions two interventions were carried out in a randomized order. Randomization was performed by a researcher outside of the measurements. These sessions were scheduled with a minimum wash-out period of 72 h in between to avoid any carry-over effects as literature states that EIH effects last from about 30 min up to a maximum of 24 h [18, 19]. Sessions consisted on the one hand of a control intervention (Control) using a 12-minute breathing exercise and on the other hand a 12-minute HIFT exercise. PPT measurements were conducted after participants had rested for 5 min (pre) and one minute after finishing the respective session (post). Participants were asked not to perform any highly intensive training 24 h prior to any intervention and not to have sore muscles, to refrain from caffeine 4 h, and not to eat 2 h before the respective sessions. When participants took any pain medication 24 h before any of the sessions or were suffering from spontaneous pain (e.g., headache) the respective intervention was rescheduled. Reporting of this study is performed according to the CONSORT checklist for randomized controlled crossover trials [20] and no changes to methods after inclusion of the first participant were done.

### Ethics

This study was conducted in accordance with the principles of good clinical and ethical practice and was approved by the local ethic committee of the University of Wuppertal (MS/AE 220203). Along with the Declaration of Helsinki, all participants gave written informed consent after being informed about the study´s protocol. This study was retrospectively registered at the German Clinical Trials Register (DRKS00034391; registration date: 4th of June 2024).

### Participants

The sample size was calculated via an a priori power analysis using G*power (Version 3.1.9.4) for a repeated measures, within-between interaction. As aerobic exercise was shown to have a large effect and dynamic resistance exercise a small effect [7], we chose to use a standardized medium effect size *f* = 0.25 along with a power of (1 − *β*) = 0.90 and an α-error probability of 0.05 resulting in a sample size of *N* = 46. Considering a dropout rate of approximately 20%, *N* = 56 participants were to be included in this study. The following inclusion criteria were considered for recruitment: Age between 18 and 35 and minimum weekly physical training amount of four hours. Participants were excluded when fulfilling one of the following criteria: Acute or chronic pain conditions, regular pain medication, joint diseases (e.g., osteoarthritis), psychological disorders, cardiovascular (e.g., hypertension), or metabolic condition (e.g., diabetes mellitus). For female participants, measurements were not performed during menstruation [21]. Participants were recruited via flyers and advertisements at the university. Participants’ characteristics are presented in Table 1.

**Table 1:** Participants’ anthropometric data and performance data of the pre-experimental test. The *p* values (*d*) refer to the unpaired t-test calculated for differences between male and female participants. Data are presented as means ± standard deviation

	Total (*N* = 50)	Male (*n* = 27)	Female (*n* = 23)	*p value (d)*
Age [years]	26.0 ± 2.7	26.1 ± 3.1	25.9 ± 2.0	0.753 (0.090)
Height [m]	1.8 ± 0.1	1.8 ± 0.1	1.7 ± 0.1	< 0.001 (2.184)
Weight [kg]	72.8 ± 12.6	80.7 ± 9.8	63.5 ± 8.5	< 0.001 (1.860)
BMI [kg/m²]	23.2 ± 2.2	24.1 ± 2.2	22.2 ± 1.8	0.002 (0.933)
Peak Power [watt]	249.8 ± 52.3	286.4 ± 35.3	206.8 ± 32.4	< 0.001 (2.338)
Rel. peak Power [watt/kg]	3.7 ± 0.6	3.8 ± 0.7	3.5 ± 0.5	0.181 (0.385)
Max. lactate [mmol]	11.3 ± 2.5	11.4 ± 2.7	11.2 ± 2.2	0.855 (0.053)
Max. HR [1/min]	184.9 ± 9.2	185.4 ± 10.0	184.3 ± 8.4	0.675 (0.120)

### Pre-experimental session

Anthropometric measurements, baseline- and stress electrocardiogram (ECG), resting and exercise blood pressure, health-related questionnaires (Physical Activity Readiness Questionnaire [22], German Pain Questionnaire [23]), as well as medical anamnesis were conducted in this pre-experimental session. PPT measurements were performed to familiarize participants with the assessment tools to avoid any confounding results due to the novelty of the measurement. Besides, a graded bicycle ergometer (Excalibur Sport 925900, Lode, Groningen, Netherlands) test up to exhaustion was conducted starting at 30 watts (W), with an increase of 40 W every three minutes. Participants were asked to remain a velocity of 80 ± 10 revolutions per minute. Heart rate (HR) was recorded using an ECG (SEMA CS-200, SCHILLER Medizintechnik Gmb, Feldkirchen, Germany). Lactate samples (20 µl) were taken from the earlobe at rest, at the end of every stage, at the time point when the exercise session was ceased, as well as one minute post exercise and subsequently analysed (Biosen S.linelab, EKF Diagnostics, Penarth, United Kingdom). Rate of perceived exertion (RPE) using the Borg Scale [24] was recorded at the end of every stage and when the exercise session was stopped.

### Exercise session

The exercise session consisted of a standardized warm-up and a HIFT program. Both were recorded beforehand, and the same video was shown to the participants. Participants were asked to imitate the exercises conducted in a high intensity manner. The warm-up routine consisted of whole-body-low-intensity and mobilization exercises, respectively, and lasted for a total of five minutes. The HIFT was designed with 40 s per exercises and 20 s rest for six different exercises, which were conducted twice, resulting in a total of 12 exercises and a total HIFT duration of 12 min (see Table 2). HR (Polar m400; Polar Electro OY, Kempele, Finland) and RPE (BORG scale) were recorded at baseline, post warm-up, in every 20 s break, and at the end of the HIFT. Before the exercise session started, participants were instructed to perform the workout in a high-intensity manner and participants were verbally motivated during the HIFT to achieve maximum effort [25].

**Table 2:** Exercises included in the high intensity functional training. Every exercise was performed in the presented order twice resulting in a total of 12 exercises (12 min)

*Exercises*:	*Duration*
4 lunges followed by one push-up (repeat)	40 s + 20 s pause
10 high knees followed by 10 shuffles (repeat)	40 s + 20 s pause
Burpees	40 s + 20 s pause
Squat jumps	40 s + 20 s pause
Skaters	40 s + 20 s pause
Plank up downs (Commandos)	40 s + 20 s pause

### Control session

Supervised deep breathing was used as a control condition as done before by van Weerdenburg et al. [26]. This active control condition was used because participants need to focus on their breathing. Hence, negative thinking and resulting psychological effects during the 12-minute control period, such as for instance ruminating, that might affect pain perception are avoided [27]. In short, participants were asked to position themselves in a relaxing and comfortable supine position. Instructions for the deep breathing session were recorded beforehand and the same recording was used for every participant by using headphones to assure a standardized procedure. Participants inhaled for four seconds and exhaled for six seconds. This was done for one minute. This produces breathing at a frequency of 0.1 Hz, corresponding to six breaths per minute. After this minute, there was a one-minute period with normal breathing. This procedure was repeated six times resulting in a total of 12 min [26]. HR was recorded and the rate of perceived relaxation (RPR) was documented on a 0–10 numeric rating scale (0 = not all relaxed, 10 = very relaxed).

### Pressure pain threshold measurements

PPT were measured using a handheld digital algometer (FPX 25 Compact Digital Algometer, Wagner Instruments, Greenwich, CT, USA) by applying pressure with a one cm^2^ rubber tip to different landmarks of the body, i.e., bilaterally to the ankle -, knee-, and elbow -joints, and the forehead as done before [28, 29]. These landmarks included the forehead (1 cm above the midpoint of the right eyebrow), and bilaterally at the joints of the elbow (lateral space below the lateral humeral epicondyle), knee (medial space at the midpoint below the medial femoral epicondyle), and ankle (lateral space between the lateral malleolus and the talus bone) [28, 29]. During these measurements, participants were positioned sitting on an examination couch with their feet suspended off the ground and their arms placed comfortably on their thighs. The use of bony landmarks offers stable and consistent reference points for measurements, thereby reducing anatomical variability and ensuring repeatability across participants and temporal points. This approach also mitigates the potential impact of muscle-related variables such as fatigue or soreness, which are known to influence PPT readings [30–32]. To avoid systematic errors, the order of these measurements was randomized in each session and this unique sequence was consistently maintained for both pre- and post-assessment within the same session. PPT measurements were performed by the same investigator. Blinding of the rater was not possible due to the participants’ exertion (e.g., heavy breathing, sweating). A cut-off value of 140 N was determined beforehand to prevent any tissue damage [33, 34]. The average value of three consecutive measurements (10 s of pause) was used for analysis. If participants did not report any pain until 140 N, a PPT value of 140 N was recorded. PPT values of each landmark are presented individually and as one mean value (PPT_total_).

### Statistics

Performance related data are presented descriptively and compared between male and female participants using an unpaired t-test. Relative mean and relative maximum HR of the HIFT/Control session were calculated by dividing the max. HR of the HIFT/Control session by the maximum HR of the pre-experimental test. This value was then multiplied by 100.

Statistical analyses were conducted on all PPT measurements taken at the seven landmarks (left ankle, right ankle, left knee, right knee, left elbow, right elbow, and forehead) individually and on the combined mean value (PPT_total_) for the different time points (pre, post) and the two interventions (HIFT, Control). All PPT data were normally distributed (tested via the Kolmogorov-Smirnov test and visual inspection of histograms and Q-Q plots) with no need for further transformation.

To evaluate potential carry-over or repeated bout effects, pre values (PPT_total_ and PPT of individual landmarks) of both interventions were compared using dependent t-tests.

The main analysis was conducted to test the 1st hypothesis. A two-way repeated-measures ANOVA was used for PPT_total_, with the factors ‘Time’ (pre, post) and ‘Intervention’ (HIFT, Control). For the individual landmarks, a three-way ANOVA was used, with the factors ‘Time’ (pre, post), ‘Intervention’ (HIFT, Control), and ‘Landmark’ (left ankle, right ankle, left knee, right knee, left elbow, right elbow, and forehead). For all ANOVA calculations, sphericity was checked (using the Mauchly test) and the Greenhouse-Geisser adjustment was used if necessary. This analysis was also used to test the 2nd hypotheses. Additionally, a one-way repeated measures ANOVA was conducted with ΔPPT of the individual landmarks (calculated by subtracting the pre value from the post value) using the factor ‘Landmark’ (left ankle, right ankle, left knee, right knee, left elbow, right elbow, and forehead).

A further analysis was performed for the stated 3rd hypothesis to detect potential differences between men and women. Here, a three-way mixed model ANOVA was calculated using PPT_total_ as the depended variable and ‘Intervention’ (HIFT, Control) and ‘Time’ (pre, post) as the within-factors. The factor ‘Sex’ (male, female) was determined as the between-factor. In addition, a two-way mixed model ANOVA was calculated using ΔPPT_total_ as the depended variable and ‘Intervention’ (HIFT, Control) as the within-factor and ‘Sex’ (male or female) as the between-factor. For these latter two analyses, homogeneity for the factor ‘Sex’ was confirmed by the Levene test. Besides, the covariate “BMI” was added to these two analyses to account for the potential influence of different BMI values observed between male and female participants.

In the case of a significant interaction effect, subsequent LSD post-hoc tests were calculated. Effect sizes are presented as partial eta-squared (*η²*_*partial*_) with values of 0.01 representing a small, 0.06 a medium, and ≥ 0.14 a large effect, respectively, and Cohen’s *d* and *d*_*z*_ for post-hoc testing with < 0.5 indicating a small effect, 0.5 to 0.8 a medium effect, and > 0.8 a large effect [35]. Statistical analyses of the data were performed using the statistics software package SPSS 27 (IBM©, Armonk, NY, United States). Data are presented as means ± standard deviation, unless otherwise marked. Differences were considered significant with *p* < 0.05.

## Results

56 participants were recruited to take part in this study. 6 participants dropped out due to personal reasons and these data were not considered in any analyses. Anthropometric and performance data of the pre-experimental test are presented in Table 1. No harms or other adverse events (e.g., falls, accidents, injuries, or fainting) occurred in this study. Performance related outcomes of the respective sessions are presented in Table 3. No carry-over effects were observed regarding PPT_total_ (*p* = 0.684) and PPT of the individual landmarks (*p* = 0.131–0.691).

**Table 3:** Participants’ performance related data of the HIFT and control session. Data are presented as means ± standard deviation. *HIFT = high intensity functional training*,* HR = heart rate*,* RPE = rate of Perceived Exertion*,* RPR = Rate of Perceived Relaxation*

	Total (*N* = 50)	Male (*n* = 27)	Female (*n* = 23)	*p value (Cohen’s d)*
HIFT max. HR [1/min]	177.7 ± 8.5	177.9 ± 8.2	177.5 ± 9.1	0.881 (0.043)
HIFT relative max. HR [%]	96.2 ± 3.6	96.0 ± 2.9	96.4 ± 4.4	0.726 (0.100)
HIFT mean HR [1/min]	168.2 ± 9.1	168.3 ± 8.4	168.0 ± 10.1	0.921 (0.028)
HIFT relative mean HR [%]	91.1 ± 4.2	90.9 ± 3.7	91.2 ± 4.8	0.746 (0.086)
Control max. HR [1/min]	65.3 ± 8.0	63.2 ± 6.5	67.8 ± 9.0	0.045 (0.583)
Control relative max. HR [%]	35.3 ± 4.0	34.1 ± 3.0	36.8 ± 4.6	0.018 (0.697)
Control mean HR [1/min]	61.7 ± 8.1	59.3 ± 6.6	64.7 ± 9.0	0.018 (0.701)
Control relative mean HR [%]	33.4 ± 4.2	32.0 ± 0.6	35.2 ± 4.6	0.006 (0.822)
HIFT max. RPE [6–20]	19.1 ± 1.2	19.5 ± 0.7	18.7 ± 1.5	0.019 (0.738)
HIFT mean RPE [6–20]	16.2 ± 1.4	16.5 ± 1.2	15.8 ± 1.5	0.100 (0.475)
Control RPR [0–10]	8.1 ± 1.3	8.3 ± 0.9	8.0 ± 1.7	0.496 (0.197)

**Fig. 1:**
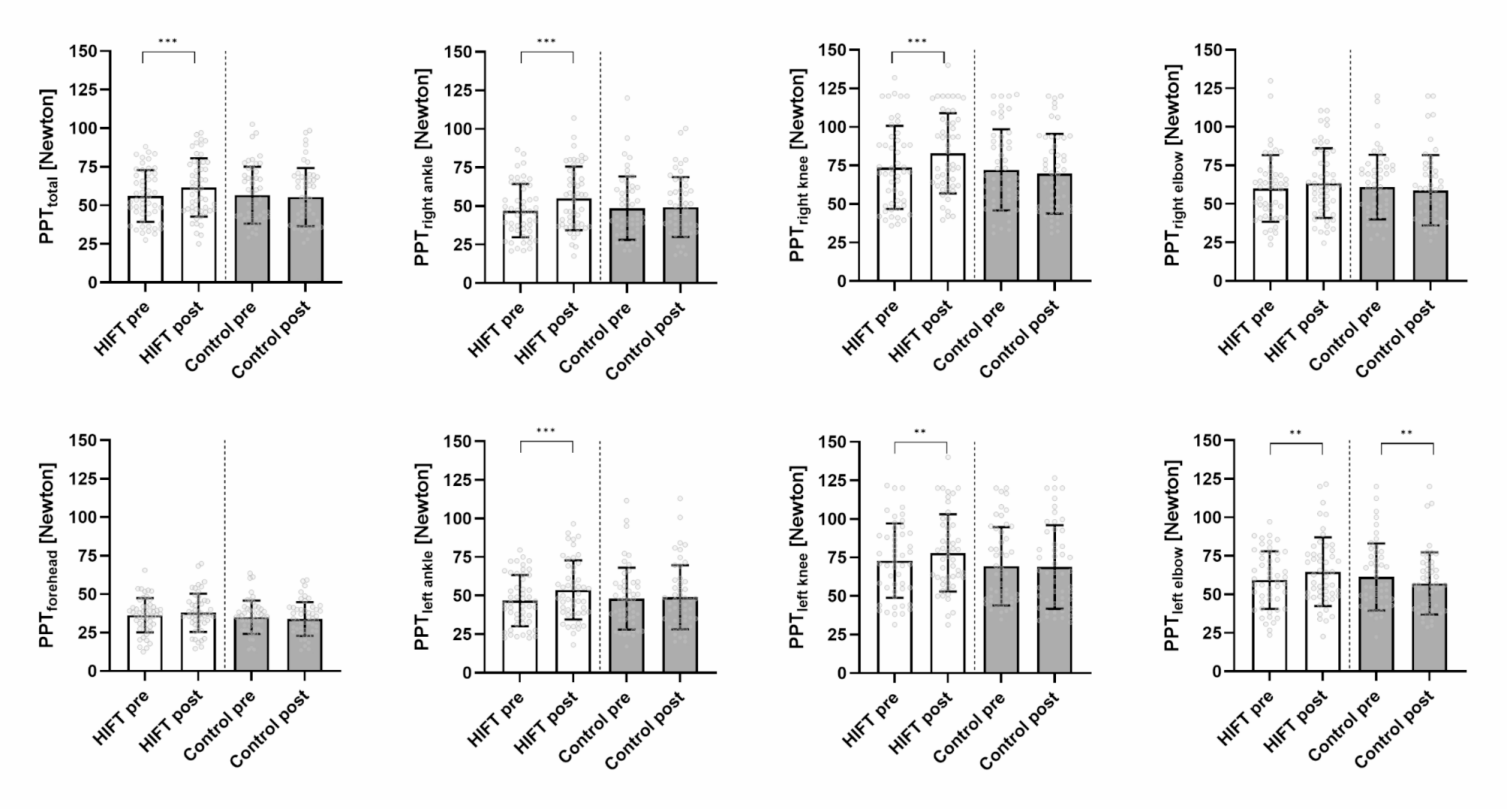
Pressure pain thresholds (PPT) in response to the HIFT and Control presented as one mean value (PPT_total_) and individual landmarks. Data are expressed as means ± standard deviation including individual data points (grey circles). Significant differences are indicated with * (*p* ≤ 0.05), ** (*p* ≤ 0.01), and *** (*p* ≤ 0.001). *HIFT = High intensity functional training*,* PPT = Pressure pain threshold*

Regarding the main 1st hypothesis, the two-way ANOVA revealed a significant ‘Intervention’ × ‘Time point’ interaction (*p* < 0.001; *η²*_*partial*_ = 0.371) for PPT_total_. The respective post-hoc test revealed hypoalgesia for PPT_total_ after the HIFT (*p* < 0.001) and no differences, but a trend toward hyperalgesia, resulting from the Control session (*p* = 0.067). Results of the three-way ANOVA reveal a significant ‘Time’ × ‘Intervention’ × ‘Landmark’ interaction effect (*p* = 0.024, *η²partial* = 0.052). The respective post-hoc test results are to be found in Fig. 1. Raw values are presented in Supplement 1 and further observed main and interaction effects and effect sizes of the three-way ANOVA are presented in Supplement 2.

**Fig. 2:**
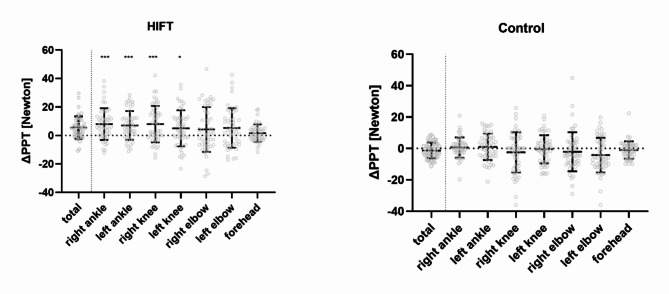
Delta (post value – pre value) pressure pain thresholds (ΔPPT) in response to the HIFT and Control of individual landmarks and represented as one mean value (PPT_total_). Data are expressed as means ± standard deviation including individual data points (grey circles). Significant differences to the forehead are indicated with * (*p* ≤ 0.05) and *** (*p* ≤ 0.001). *HIFT = High intensity functional training*,* PPT = Pressure pain threshold*

Regarding the 2nd hypothesis, the one-way ANOVA performed for the HIFT session revealed significant differences between the landmarks (*p* = 0.034; *η²*_*partial*_ = 0.050) with higher values observed for ΔPPT of the lower extremity landmarks compared to the forehead (see Fig. 2). No differences between landmarks were observed in the Control session (*p* = 0.080; *η²*_*partial*_ = 0.040).

**Fig. 3:**
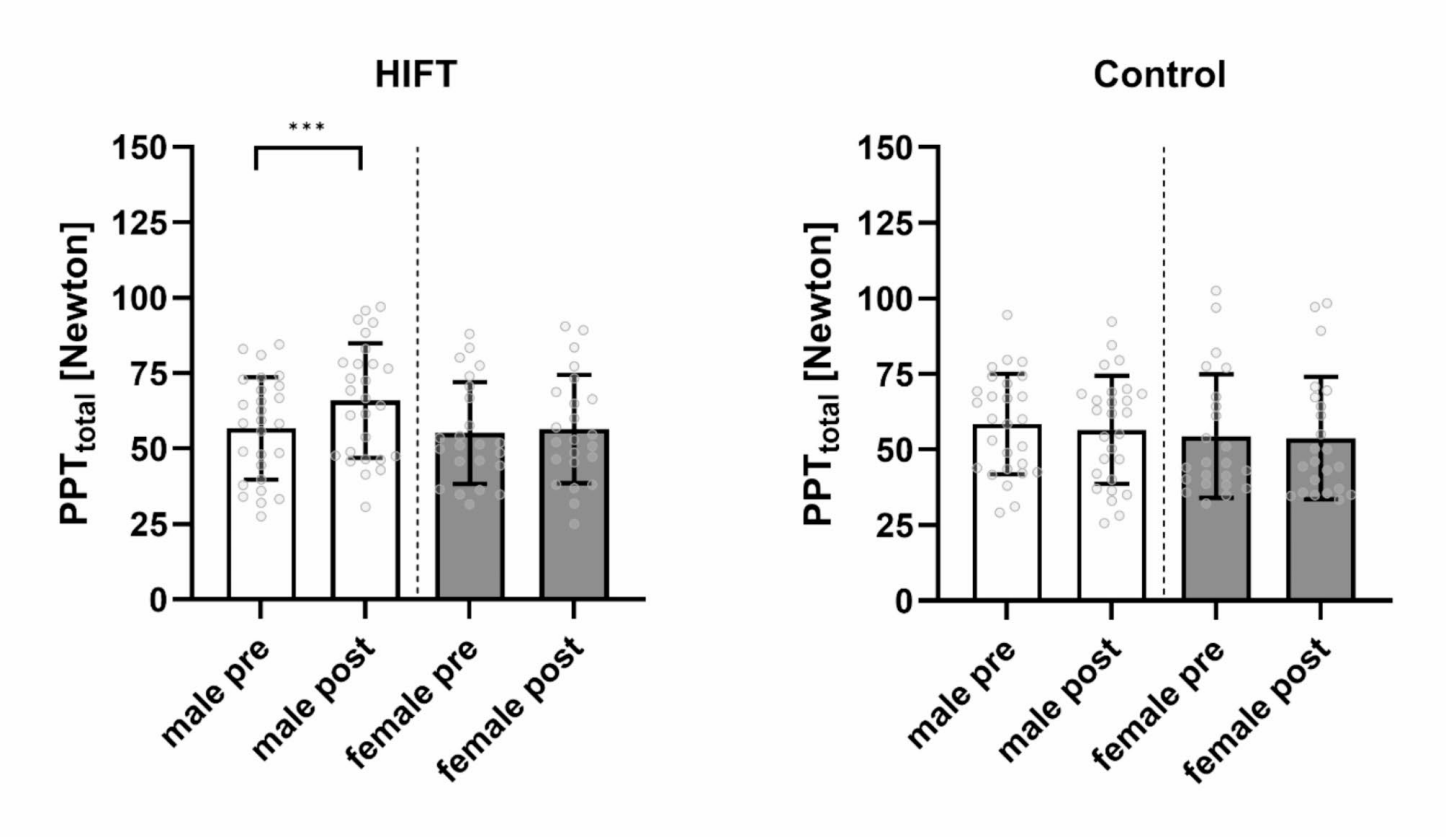
Pressure pain thresholds (PPT) in response to the HIFT and Control presented as one mean value (PPT_total_) separately presented for male and female participants. Data are expressed as means ± standard deviation including individual data points (grey circles). Significant differences are indicated with *** (*p* ≤ 0.001). *HIFT = High intensity functional training*,* PPT = Pressure pain threshold*

Regarding the 3rd hypothesis, the three-way mixed model ANOVA to detect potential differences between men (*n* = 27) and women (*n* = 23) revealed a significant ‘Intervention’ × ‘Time’ × ‘Sex’ interaction effect (*p* < 0.001, *η²partial* = 0.257). Subsequently calculated post-hoc tests revealed that male participants showed higher PPT_total_ (hypoalgesia) values following the HIFT session (*p* < 0.001), while no differences were observed in female participants (*p* = 0.363). The Control session revealed a trend towards hyperalgesia in males (*p* = 0.055), but no difference in females (*p* = 0.531; see Fig. 3). Further observed main and interaction effects and effect sizes of the three-way ANOVA are presented in Supplement 3. The covariate “BMI” did not significantly adjust the results observed (*p* = 0.369). A significant ‘Intervention’ × ‘Time’ × ‘Sex’ interaction effect is still observed for the three-way mixed model ANCOVA (*p* = 0.002, *η²partial* = 0.195).

Besides, the two-way mixed model ANOVA revealed a significant ‘Intervention’ × ‘Sex’ effect (*p* < 0.001, *η²partial* = 0.252) and a significant ‘Intervention’ (*p* < 0.001, *η²partial* = 0.411) and ‘Sex’ (*p* = 0.010, *η²partial* = 0.131) effect for ΔPPT_total_. Subsequently calculated post-hoc test showed that male (9.21 ± 7.79 N) participants exerted a larger degree of hypoalgesia compared to female participants (1.33 ± 5.28 N; *p* < 0.001) following the HIFT session (data not shown). The covariate “BMI” did not significantly adjust the results observed (*p* = 0.952). A significant ‘Intervention’ × ‘Sex’ interaction effect is still observed for the two-way mixed model ANCOVA (*p* = 0.002, *η²partial* = 0.194).

## Discussion

This study evaluated effects of a single HIFT session on EIH compared to a control session. Results confirm the 1st hypothesis as the HIFT session resulted in global hypoalgesia indicated by higher PPT_total_ values observed after the training, while no such effects were observed after the control session. Further, and in the light of the 2nd hypothesis, the HIFT resulted in hypoalgesia observed at all landmarks except for the right elbow and the forehead. Regarding ΔPPT values, ΔPPT of the lower extremity landmarks were higher compared to the forehead. Interesting findings were observed regarding the 3rd hypothesis. Here, hypoalgesia was observed in male participants following the HIFT, while female participants did not reveal such hypoalgesia.

The HIFT was designed to be highly intensive and to consist of different multimodal and functional exercises. Participants reached an average HR and maximum HR throughout the HIFT that was above 90% and 95%, respectively, of their individual maximum HR determined in the pre-experimental test. Further, participants perceived this HIFT as very hard as observed in maximum RPE values of above 19, equalling a description between “very hard” and “very, very hard” [24].

Regarding hypoalgesic effects and in the light of the 1st hypothesis, current literature mainly focuses on unimodal and constant load aerobic exercises, such as cycling and running, and its hypoalgesic effects [7]. Within this aerobic modality it is believed that hypoalgesia occurs more robustly the higher the intensity is [5, 28, 36–38]. For instance, Naugle et al. showed that 20 min of vigorous cycling exercise at 70% of HR reserve increased PPT whereas PPT was unaltered after moderate exercise at 50% of HR reserve [37]. Another study by Vaegter et al. also revealed that a high intensity bicycle exercise at a calculated intensity of 75% VO_2_max led to a larger EIH response compared to a low intensity exercise conducted at 50% VO_2_max [36]. Most likely, the present HIFT involved a substantial aerobic component, but as the exercises were performed for 40 s and with very high intensity followed by a 20-second recovery period, there was also a large anaerobic component involved in the HIFT. Previous research dealing with anaerobic exercises show that these exercise types (e.g., 90-second all-out isokinetic cycling [29], a ‘Wingate Anaerobic Test’ for 30 s [39], or 60-second all-out rowing [28]) result in hypoalgesia. Therefore and also in the light of the results of the present study, high intensity exercises along with a high anaerobic demand are most likely to induce EIH.

Yet, hypoalgesic effects resulting from dynamic resistance exercise are less explored and the current literature indicates that dynamic resistance exercise only has small effects on hypoalgesia as indicated by a recent meta-analysis [7]. One major difference between conventional dynamic resistance training and HIFT is that the cardiovascular strain is much higher during HIFT when considering the HR profile. For instance, one study conducted a conventional strength training consisting of lifting three sets of 10 repetitions at 75% of an individual’s one repetition maximum. This training led to HR of around 100 beats/min [40]. Another study conducted a 40-minute dynamic circuit resistance training at 60% of the individuals’ one repetition maximum to evaluate differences in pain sensitivity. This training led to HR of around 150 beats/min and lactate concentrations of around 10 mmol/l. PPT were measured at the dorsal side of the non-dominant hand at the base of the skin web between the thumb and index finger. Results reveal no difference in PPT from pre to post exercise [41]. Contrastingly, the results presented herein show that a HIFT session, which consisted of dynamic multimodal exercises using the own body weight and no equipment, induces hypoalgesia. The exercises can be characterized as incorporating dynamic resistance exercises, which at the same time are highly demanding for the cardiovascular system, as observed in the very high HR and RPE values. Hence, the combination of muscular and cardiovascular strain during the HIFT might be responsible for inducing hypoalgesia. In the present study, no blood pressure responses were measured. Yet, is known that heavy resistance exercise can result in intraarterial systolic and diastolic blood pressures of up to 320/250 mmHg, especially when a Valsalva manoeuvre is performed [42]. However, blood pressure increases are more moderate during and following HIFT with blood pressure increases to 154/77 and 140/71 in male and female participants, respectively, following a 15-minute HIFT session [43]. Reduced pain sensitivity is most likely induced by the release of analgetic endogenous opioid-related substances expressed centrally in the nervous system. Moreover, these substances are released more locally close to the contractile musculature attenuating nociceptive signalling [44, 45]. It is therefore proposed that hypoalgesia is highest at body landmarks close to the working musculature and less pronounced at remote landmarks [19].

This assumption was tested as part of the 2nd hypothesis. Results presented herein reveal that hypoalgesia occurred primarily at the ankle and knee joints. The used exercises in the HIFT focussed mainly on the lower extremity musculature (i.e., lunges, high knees, burpees, squad jumps, and skaters). Contrastingly, no hypoalgesia was observed at the forehead and right elbow. However, hypoalgesia also occurred at the left elbow. The reasons explaining these side differences at the elbows remain elusive and cannot be sufficiently explained. Yet, hypoalgesic effects may be less pronounced and may occur less consistently depending on the distance to the primary exercising body parts and musculature [36, 46]. Besides, it might be possible that participants conducted some exercises (i.e., push-up, commandos) with a more pronounced activity of the dominant arm, which resulted in divergent hypoalgesic effects at the elbows. Yet, no information on the handedness of the participants were recorded, which might have had provided some explanations for this observation. The phenomenon that no effects were present at the forehead was also observed in another study, in which participants conducted differently intensive bicycle ergometer sessions for 30 min. In this study, hypoalgesia was also observed at the elbows, but not at the forehead [5]. Yet, a maximal “all-out” rowing exercise of about 60 s as well as “all-out” isokinetic bicycle exercises of 15 and 90 s, respectively, resulted in global hypoalgesia also observed at the forehead [28, 29].

Interestingly and in the light of the 3rd hypothesis, only male participants revealed hypoalgesia. Reasons explaining these findings remain elusive. Male and female participants exerted similar performance levels (i.e., Watt/kg) excluding differences in pain processing or perception due to fitness levels [47]. BMI values were different between male (24.1 ± 2.2) and female (22.2 ± 1.8) participants, even though both being in the normal weight range, which might influence EIH responses due to different fitness levels regrading muscle and/or fat mass. Yet, this differentiation in body composition was not considered in this study. The covariate analyses reveal that the participants’ BMI did not adjust the results. Both relative average and maximum HR observed during the HIFT did not differ between men and women. Yet, maximum RPE values (but not average RPE) are slightly but significantly higher in men (19.5 ± 0.7 vs. 18.7 ± 1.5; *d* = 0.738). Therefore, the observed divergent results regarding EIH might in part be explained by different subjectively perceived effort but not by objectively measured exertion. While direct comparisons between men and women regarding EIH are limited in the literature, the available research suggests that men and women experience EIH as observed in studies including men and women [21, 48, 49] as well as in studies including only women [50]. However, some studies reveal specific differences in EIH between men and women. In two studies, isometric exercises were shown to result in EIH in women but not in men [9, 10], while research also shows that no sex-dependent differences in EIH occur after isometric exercise [11]. Yet, further research directly comparing sex-specific differences in EIH across various conditions is necessary for a definitive understanding.

In contrast to the HIFT, the control session did not result in hypoalgesia. The control session was perceived as relaxing with the degree of relaxion being at about 8 out of 10. Previous research demonstrated that slow, deep breathing procedures can result to lower ratings of heat pain intensity ratings [51], increased thermal pain thresholds [52] and hypoalgesic suprathreshold electrical stimulations [53]. In the present study, no such effects were observed for mechanical PPT.

### Strengths and limitations

The main strengths of this study are that a 12-minute body weight HIFT protocol was developed that led to average HR of over 90% and maximum HR of over 95% of the participants’ individual HR, respectively. Consequently, this program can be used as a home-based HIFT (e.g., during public lockdowns), which does not need any additional equipment, to induce hypoalgesia. Yet, there are some limitations to this research study. It should be noted that our study was conducted only with young and healthy adults who were physically active. Hence, results are not transferable to any clinical populations. Due to the intentionally set very high intensity, this HIFT program and the used exercises as well as the results observed might also not be transferable to very untrained people, as the experienced high exertion resulting from this training might result in a loss of motivation and pleasure [54, 55]. Besides, it is not suitable for chronic pain patients in clinical settings suffering from severe pain due to psychological factors associated, such as kinesiophobia [56] or catastrophising [57]. Further, PPT were measured solely at bony landmarks, limiting their comparability with studies that use muscular landmarks for PPT assessment. Future studies should also aim to evaluate the difference response to exercise when using bony and muscular landmarks for PPT measurements. Important information regarding, for instance the use of contraceptives or menstrual cycle phase, were not documented. Further, due to the high intensity of the HIFT and the nature of the control session, blinding of the rater for pain measurement was not possible.

### Future directions

Future research should aim to compare further pain physiological effects resulting from trending and/or innovative training methods. HIFT should be compared to traditional training modalities such as conventional isometric or dynamic resistance training or aerobic training modalities in terms of its pain reducing potentials. Besides, the time span of hypoalgesic effects after exercise should be investigated in future studies, as this investigation (and most other publications) only tested immediate effects. Moreover, future research should focus on sex-specific research questions in the context of EIH, since these results, as well as other previously published studies, might hint to the fact that differences in pain processing between men and women can exist. Here, the use of contraceptives should be controlled and the menstrual cycle phase, when interventions (i.e., exercise and controls sessions) are performed, should be carefully considered [58]. Future research should also explore the clinical possibilities of high or higher intensity exercise regimens (such as HIFT) as a therapeutic tool to manage specific pain conditions. This kind of exercise may be suitable for people experiencing mild and/or intermittent forms of nonspecific pain, such as for instance nonspecific chronic low back pain, which was shown to increase in prevalence and intensity during public lockdowns [59, 60]. It is important to note that this was not assessed in the study presented herein and warrants further investigation in future research.

## Conclusion

This study for the first time presents that a short 12-minute HIFT session in which participants achieve a mean HR of > 90% of their individual maximum HR leads to EIH. On a physiological level, the study shows that the combination of strength and aerobically demanding exercises going along with a high cardiovascular and muscular demand induces hypoalgesia. This reduced pain sensitivity occurs predominantly at lower extremity body parts while no changes were observed at the forehead and right elbow. An interesting finding is that hypoalgesia was only present in male and not in female participants suggesting sex-related differences in the context of EIH. The HIFT training method can be easily conducted at home as no additional material (e.g., dumbbells, free weights, etc.) are needed. Especially during times when public sport facilities are closed or no group training is possible (e.g., during public lockdowns), the proposed HIFT session can be easily used to modulate pain sensitivity.

### Electronic supplementary material

Below is the link to the electronic supplementary material.

Supplementary Material 1

Supplementary Material 2

## Data Availability

Data is provided within the manuscript or supplementary information files.

## Abbreviations

HIFT: High-Intensity Functional Training
PPT: Pressure Pain Threshold
HIIT: High-Intensity Interval Training
CONSORT: Consolidated Standards of Reporting Trials
ECG: Electrocardiogram
HR: Heart Rate
RPE: Rating of Perceived Exertion
RPR: Rate of Perceived Relaxation
