# Supplementary material for: Exercise induced hypoalgesia after a high intensity functional training: a randomized controlled crossover study
Source: BMC Sports Sci Med Rehabil. 2024 Aug 28;16:182. doi: 10.1186/s13102-024-00969-4 (PMC11351546; doi:10.1186/s13102-024-00969-4)
Supplement: Supplementary file 1 — Supplementary Material 1 [file 13102_2024_969_MOESM1_ESM.docx]

**Supplement 1**: Results of PPT measurements pre and post HIFT and Control intervention. Data are presented as means ± SD. Effect size [*d_z_*] is presented for the paired samples of the respective landmark and intervention. *PPT = Pressure pain threshold measured via pressure algometer; HIFT= High intensity functional training.*

|  | HIFT | | CONTROL | |
| --- | --- | --- | --- | --- |
| PPT [Newton] | | | | |
|  | **Mean ±SD** | **effect size [*d_z_*]** | **Mean ±SD** | **Effect size [*d_z_*]** |
| Pre PPT_total_ | 56.0 ± 16.8 |  | 56.6 ± 18.4 |  |
| Post PPT_total_ | 61.6 ± 19.0 | -0.710 | 55.3 ± 18.9 | 0.265 |
| Pre PPT_right ankle_ | 46.7 ± 17.3 |  | 48.6 ± 20.5 |  |
| Post PPT_right ankle_ | 54.9 ± 16.5 | -0.712 | 49.3 ± 19.4 | -0.101 |
| Pre PPT_left ankle_ | 46.7 ± 16.5 |  | 48.0 ± 20.1 |  |
| Post PPT_left ankle_ | 53.7 ± 19.1 | -0.719 | 49.0 ± 20.7 | -0.116. |
| Pre PPT_right knee_ | 73.7 ± 26.9 |  | 72.1 ± 26.3 |  |
| Post PPT_right knee_ | 83.0 ± 26.0 | -0.727 | 69.7 ± 25.9 | 0.193 |
| Pre PPT_left knee_ | 72.9 ± 24.1 |  | 69.4 ± 25.4 |  |
| Post PPT_left knee_ | 78.0 ± 25.0 | -0.398 | 68.8 ± 27.1 | 0.062 |
| Pre PPT_right elbow_ | 60.1 ± 21.6 |  | 61.0 ± 20.9 |  |
| Post PPT_right elbow_ | 63.5 ± 22.5 | -0.217 | 58.9 ± 22.9 | 0.168 |
| Pre PPT_left elbow_ | 59.3 ± 18.7 |  | 61.3 ± 21.8 |  |
| Post PPT_left elbow_ | 64.7 ± 22.3 | -0.391 | 57.1 ± 20.2 | 0.386 |
| Pre PPT_forehead_ | 36.3 ± 11.2 |  | 34.5 ±10.8 |  |
| Post PPT_forehead_ | 37.9 ± 12.5 | -0.270 | 33.9 ±11.0 | 0.198 |

**Supplement 2:** Results of the three-way ANOVA for the dependent variable PPT with the factors ‘Intervention’ (HIFT, Control), ‘Time’ (pre, post), and ‘Landmark’ (forehead, sternum, knee joints, ankle joints, and elbows).

| Main and interaction effects | *p*-value | η²partial |
| --- | --- | --- |
| ‘Time’ × ‘Intervention’ × ‘Landmark’ | 0.024 | 0.052 |
| ‘Intervention’ × ‘Time’ | < 0.001 | 0.368 |
| ‘Intervention’ × ‘Landmark’ | 0.028 | 0.053 |
| ‘Landmark’ × ‘Time’ | 0.019 | 0.058 |
| ‘Landmark’ | < 0.001 | 0.682 |
| ‘Time’ | 0.002 | 0.179 |
| ‘Intervention’ | 0.013 | 0.120 |

*η²_partial_= partial eta-squared*

**Supplement 3:** Results of the three-way ANOVA for the dependent variable PPT_total_ with the factors ‘Intervention’ (HIFT, Control), ‘Time’ (pre, post), and ‘Sex’ (male, female).

| Main and interaction effects | *p*-value | η²partial |
| --- | --- | --- |
| ‘Time’ × ‘Intervention’ × ‘Sex | < 0.001 | 0.257 |
| ‘Intervention’ × ‘Time’ | < 0.001 | 0.414 |
| ‘Intervention’ × ‘Sex | 0.450 | 0.012 |
| ‘Sex’ × ‘Time’ | 0.011 | 0.127 |
| ‘Sex’ | 0.375 | 0.016 |
| ‘Time’ | 0.003 | 0.172 |
| ‘Intervention’ | 0.053 | 0.076 |

*η²_partial_= partial eta-squared*
