# Supplementary material for: Exercise induced hypoalgesia after a high intensity functional training: a randomized controlled crossover study
Source: BMC Sports Sci Med Rehabil. 2024 Aug 28;16:182. doi: 10.1186/s13102-024-00969-4 (PMC11351546; doi:10.1186/s13102-024-00969-4)
Supplement: Supplementary file 2 — Supplementary Material 2 [file 13102_2024_969_MOESM2_ESM.docx]

CONSORT checklist for randomized controlled crossover trials

| Section/Topic | Item No | Checklist item | Reported on page No |
| --- | --- | --- | --- |
| Title and abstract | | | |
|  | 1a | Identification as a randomised crossover trial in the title | See Title |
|  | 1b | Specify a crossover design and report all information outlined | See Abstract |
|  | | | |
| Background and objectives | 2a | Scientific background and explanation of rationale | 3 |
|  | 2b | Specific objectives or hypotheses | 3 |
|  | | | |
| Trial design | 3a | Rationale for a crossover design. Description of the design features including allocation ratio,  especially the number and duration of periods, duration of washout period, and consideration of  carry over effect | 4 & 7 |
|  | 3b | Important changes to methods after trial commencement (such as eligibility criteria), with reasons | 4 |
| Participants | 4a | Eligibility criteria for participants | 4 |
|  | 4b | Settings and locations where the data were collected | 4 |
| Interventions | 5 | The interventions with sufficient details to allow replication, including how and when they were actually  administered | 5f |
| Outcomes | 6a | Completely defined prespecified primary and secondary outcome measures, including how and when they were assessed | 5f |
|  | 6b | Any changes to trial outcomes after the trial commenced, with reasons | 4 |
| Sample size | 7a | How sample size was determined, accounting for within participant variability | 4 |
|  | 7b | When applicable, explanation of any interim analyses and stopping guidelines | n.a. |
| Randomisation: |  |  |  |
| Sequence generation | 8a | Method used to generate the random allocation sequence | 4 |
|  | 8b | Type of randomisation; details of any restriction (such as blocking and block size) | 4 |
| Allocation concealment mechanism | 9 | Mechanism used to implement the random allocation sequence (such as sequentially numbered containers), describing any steps taken to conceal the sequence until interventions were assigned | 4 |
| Implementation | 10 | Who generated the random allocation sequence, who enrolled participants, and who assigned participants to interventions | 4 |
| Blinding | 11a | If done, who was blinded after assignment to interventions (for example, participants, care providers, those assessing outcomes) and how | No blinding was performed |
|  | 11b | If relevant, description of the similarity of interventions | n.a. |
| Statistical methods | 12a | Statistical methods used to compare groups for primary and secondary outcomes which are appropriate for  crossover design (that is, based on within participant comparison) | 7 |
|  | 12b | Methods for additional analyses, such as subgroup analyses and adjusted analyses | 7 |
| Results | | | |
| Participant flow (a diagram is strongly recommended) | 13a | The numbers of participants who were randomly assigned, received intended treatment, and were analysed  for the primary outcome, separately for each sequence and period | 8 |
|  | 13b | No of participants excluded at each stage, with reasons, separately for each sequence and period | 8 |
| Recruitment | 14a | Dates defining the periods of recruitment and follow-up | 4 |
|  | 14b | Why the trial ended or was stopped | n.a. |
| Baseline data | 15 | A table showing baseline demographic and clinical characteristics by sequence and period | Table 1 |
| Numbers analysed | 16 | Number of participants (denominator) included in each analysis and whether the analysis was by original  assigned groups | 8, 10 |
| Outcomes and estimation | 17a | For each primary and secondary outcome, results including estimated effect size and its precision (such as  95% confidence interval) should be based on within participant comparisons.¶ In addition, results for each  intervention in each period are recommended | Table 1 & 3, Figure 1-3, Supplement 1 |
|  | 17b | For binary outcomes, presentation of both absolute and relative effect sizes is recommended | n.a. |
| Ancillary analyses | 18 | Results of any other analyses performed, including subgroup analyses and adjusted analyses, distinguishing pre-specified from exploratory | n.a. |
| Harms | 19 | Describe all important harms or untended effects in a way that accounts for the design (for specific  guidance, see CONSORT for harms) | 8 |
| Discussion | | | |
| Limitations | 20 | Trial limitations, addressing sources of potential bias, imprecision, and if relevant, multiplicity of analyses.  Consider potential carry over effects | 13f |
| Generalisability | 21 | Generalisability (external validity, applicability) of the trial findings | 11ff |
| Interpretation | 22 | Interpretation consistent with results, balancing benefits and harms, and considering other relevant evidence | 11ff |
| Other information | | |  |
| Registration | 23 | Registration number and name of trial registry | 4 |
| Protocol | 24 | Where the full trial protocol can be accessed, if available | N.a. |
| Funding | 25 | Sources of funding and other support (such as supply of drugs), role of funders | N.a. |

*We strongly recommend reading this statement in conjunction with the CONSORT 2010 Explanation and Elaboration for important clarifications on all the items. If relevant, we also recommend reading CONSORT extensions for cluster randomised trials, non-inferiority and equivalence trials, non-pharmacological treatments, herbal interventions, and pragmatic trials. Additional extensions are forthcoming: for those and for up to date references relevant to this checklist, see [www.consort-statement.org](http://www.consort-statement.org).
